# Outbreak of *Leptospira borgpetersenii* Serogroup Sejroe Infection in Kennel: The Role of Dogs as Sentinel in Specific Environments

**DOI:** 10.3390/ijerph19073906

**Published:** 2022-03-25

**Authors:** Andrea Balboni, Elisa Mazzotta, Maria Beatrice Boniotti, Cristina Bertasio, Laura Bellinati, Laura Lucchese, Mara Battilani, Letizia Ceglie, Silvia Marchione, Giulio Esposito, Alda Natale

**Affiliations:** 1 Department of Veterinary Medical Sciences, Alma Mater Studiorum—University of Bologna, 40064 Bologna, Italy; a.balboni@unibo.it (A.B.); mara.battilani@unibo.it (M.B.); 2 Istituto Zooprofilattico Sperimentale delle Venezie, Viale dell’Università, 35020 Legnaro, Italy; emazzotta@izsvenezie.it (E.M.); lbellinati@izsvenezie.it (L.B.); llucchese@izsvenezie.it (L.L.); lceglie@izsvenezie.it (L.C.); smarchione@izsvenezie.it (S.M.); 3 Italian Reference Centre for Animal Leptospirosis, Istituto Zooprofilattico Sperimentale della Lombardia e dell’Emilia Romagna “Bruno Ubertini”, 25121 Brescia, Italy; mariabeatrice.boniotti@izsler.it (M.B.B.); cristina.bertasio@izsler.it (C.B.); 4 Unità Operativa Complessa Veterinaria A e C Sanità Animale e Igiene degli Allevamenti e delle Produzioni Zootecniche, Azienda USL di Bologna, 40124 Bologna, Italy; g.esposito@ausl.bologna.it

**Keywords:** dog, kennel, Italy, *Leptospira*, outbreak, Sejroe

## Abstract

Kennels may represent high-risk environments for the diffusion of *Leptospira* infection in dogs and consequently a threat to public health. This study describes an outbreak of *Leptospira* infection in a kennel in Italy in 2020, both with clinically ill and asymptomatic dogs. Fifty-nine dogs, including three ill dogs, were tested for *Leptospira* spp. infection by the microscopic agglutination test (MAT) and real-time qPCR. Multi-locus sequence typing (MLST) analysis was used to genotype the identified leptospires. Thirty of the fifty-nine (50.9%) dogs had MAT titer and/or molecular positivity indicative of *Leptospira* infection. Twenty-two of the fifty-nine (37.3%) dogs exhibited seropositivity against at least one serovar belonging to the Sejroe serogroup, and MLST analysis identified *L. borgpetersenii* serogroup Sejroe (*Leptospira* ST155) as responsible for the outbreak. Up to now, Sejroe serogroup infection was sporadically reported in dogs. The extension of the MAT antigen panel to several serovars belonging to the serogroup Sejroe could be useful in the diagnosis of canine leptospirosis. Dogs may serve as sentinel of leptospires in specific environments, and surveillance of *Leptospira* infection in kennels is strongly recommended even when the correct vaccine prophylaxis is administered, because the vaccines currently available are not able to protect from all of the serogroups.

## 1. Introduction

Leptospirosis is a worldwide zoonosis caused by infection with pathogenic Gram-negative and highly motile spirochete bacteria of the genus *Leptospira*. Pathogenic leptospires infect different mammalians among wild and domestic animals, including humans. Susceptible hosts become infected by contact of intact mucous membranes or abraded skin with infected urine or urine-contaminated fomite such as water, soil, food, or equipment [1].

The maintenance or reservoir host plays a very important epidemiological role and is defined as an animal capable of acting as a natural source of infection for specific *Leptospira* serovars [2]. Reservoir hosts often do not exhibit clinical signs of disease, but leptospires colonize the tubular lumen of the kidney and are persistently excreted via urine, contaminating the environment [3]. Differently, incidental hosts can develop acute and severe disease [2,3].

Canine leptospirosis is caused by several *Leptospira* serovars with variable geographic distribution and reporting a wide range of clinical manifestations, from subclinical to severe [1,3]. The main serogroups to which dogs in Europe are apparently exposed are Icterohaemorrhagiae, Grippotyphosa, Australis, Sejroe, and Canicola [4]. Among these serogroups, dogs can represent reservoir hosts for Canicola and potentially Australis, while the serovars belonging to the other serogroups are maintained by a large variety of hosts, in particular rodents, mice, and rats [4]. Consequently, the epidemiology of canine leptospirosis may vary by geographic area and change over time in relation to the spread of maintenance hosts and vaccination.

Vaccination is the method of choice to prevent leptospirosis in dogs, although the evoked immune protection is primarily serovar-specific and partially serogroup-specific. In Europe and Italy, in the last 60 years, the wide use of commercial bivalent vaccines against Canicola and Icterohaemorrhagiae serovars has reduced the homologous infections due to Canicola and has controlled the clinical forms due to Icterohaemorrhagiae in vaccinated dogs [5,6,7]. Consequently, Grippotyphosa and Australis emerged as responsible for clinical syndromes in dogs. To achieve the vaccine protection in dogs, new trivalent or tetravalent vaccines containing antigens from up to four different serovars belonging to Canicola, Icterohaemorrhagiae, Australis, and Grippotyphosa serogroups have been licensed in Europe [3,4,8,9].

In Italy, recent surveys conducted by the microscopic agglutination test (MAT) on dogs reported seroprevalence values ranging from less than 10% to over 20%. The results are influenced by the geographic area investigated, the inclusion of symptomatic or healthy subjects, the vaccine responses, the panel of serovars adopted for the assay, and the variability of the cut-off titer adopted to identify positive subjects [7,10,11,12]. In these studies, the most prevalent serogroups were Icterohaemorrhagiae, Grippotyphosa, Australis, and Canicola, and a low number of positive sera were also observed for Pomona and Sejroe serogroups.

Kennels may represent high-risk environments for the diffusion of leptospiral infection in dogs, as facilitated by the close contact of a large number of dogs kept in a relatively small area, with potentially poor hygiene standards and the possible presence of mice and rats [7]. Consequently, an outbreak of leptospirosis in a kennel can represent a severe threat to public health, as numerous shelter operators, volunteers, and visitors can be exposed to the pathogen.

In this study, an outbreak of *Leptospira* infection involving dogs both with clinical manifestations and asymptomatic infection in a kennel in Italy in 2020 is described. An unusual *Leptospira* variant was identified as responsible for the infection in kennel dogs and implications for veterinary medicine and public health are discussed from a One-Health point of view.

## 2. Materials and Methods

### 2.1. Study Design, Population, and Sampling

This was a retrospective study describing a leptospirosis outbreak in a kennel in Italy in 2020. The kennel is located near a small stream and surrounded by brush and trees. At the time of the infection, it consisted of 68 boxes and housed 78 dogs. The kennel was served by a private well for water and the presence of mice and rats was reported between the dog boxes and the service rooms.

At the end of October 2020, three dogs showed clinical manifestations referable to leptospirosis. One dog (ID: 57878) showed clinical signs of acute disease related to the presence of acute kidney injury (AKI) and liver impairment, such as anorexia, depression, lethargy, fever, jaundice, polyuria, polydipsia, and vomiting. The other two dogs (ID: 58043 and 60311) showed less severe clinical signs, such as anorexia, depression, lethargy, and mild jaundice. No history of previous cases of canine leptospirosis in this kennel was reported. The 3 dogs showing clinical signs referable to leptospirosis and another 56 apparently healthy dogs were enrolled in the study. The remaining 19 dogs were excluded because they were aggressive and could not be sampled without sedation. The sampled dogs came from all areas of the kennel facility and were tested for *Leptospira* spp. infection by serologic and molecular assays. The samples that tested positive for leptospiral DNA were subjected to multi-locus sequence typing (MLST) analysis in order to genotype the detected leptospires.

From dogs included in the study, urine samples were collected by spontaneous voiding and blood sampling was carried out by venipuncture. Urine and/or EDTA-treated blood samples were used for molecular analyses. Serum samples were used for the serologic assay. Signalment, anamnestic, and vaccination data were retrieved from kennel and medical records. Vaccination status was compared to international guidelines for the vaccination of dogs [13]. The actions taken to control and treat infections were prescribed by the territorial veterinary health authority. To evaluate possible environmental sources of infections, samples of 250 mL of water from the well that served the kennel were collected from four different areas of the kennel and tested for the presence of pathogenic *Leptospira* DNA. All samples were stored at 4 °C, for a maximum of 24 h, until analysis.

Both the urine and blood sampling were performed for clinical and diagnostic purposes, in agreement with the legal manager of the kennel. The territorial health authority tested the naturally infected dogs as the health service protocols indicate for management of the *Leptospira* outbreaks. As all sampling activity was independent of the study, no separate ethical approval was required. However, for preserving the dogs’ welfare, accurate attentions were provided to minimize the discomfort of the animals during the sampling procedures.

### 2.2. Microscopic Agglutination Test (MAT)

Dog serum samples were tested for antibodies against pathogenic *Leptospira* using the microscopic agglutination test (MAT) following the Office International des Epizooties (OIE) method [14]. The antigen panel included 8 serogroups and 11 serovars distributed by the Italian Reference Center for Animal Leptospirosis as antigens in the routine diagnostic MAT (Table 1). Serum samples were pretested at the final dilution of 1:100. Serum samples with 50% agglutination were retested to determine an endpoint using dilutions of serum beginning at 1:100 through to 1:6400. Serum samples with the widely accepted minimum significant titer of 1:100 (reciprocal of the final dilution of serum with 50% agglutination) were assessed as positive. Positive titers ≥ 1:100 against non-vaccine serogroups or ≥1:800 against vaccine serogroups (Canicola and Icterohaemorrhagiae for bivalent vaccine, and Canicola, Icterohaemorrhagiae, Australis, and Grippotyphosa for tetravalent vaccine) were recognized as of potential infectious origin, while positive titers < 1:800 against vaccine serogroups only were recognized as of potential vaccine origin.

### 2.3. Sample Pretreatments and DNA Extraction

Urine, EDTA-treated blood, and well water samples were tested for the presence of pathogenic *Leptospira* DNA.

Urine samples were subjected to a centrifugation step before extraction, in order to concentrate the leptospires and increase the yield of DNA. Therefore, 2 mL of urine was centrifuged at 12,000× *g* for 20 min at 4 °C, and the pellet was resuspended in 200 µL of sterile PBS.

A filtration step was applied to the water samples as well to concentrate them, according to a previous assay performed to investigate the filter capacity. Briefly, 250 mL of water was contaminated with 2.5 mL of *L. interrogans* serovar *Icterohaemorrhagiae* culture and filtered through an individual 0.22 µm pore-sized nitrocellulose filter (Merck KGaA, Darmstadt, Germany) placed on a Polycarbonate Filter Holder (Sartorius AG, Gottinga, Germany) at −30 mbar vacuum pressure [15]. Both the filter and filtered water were tested for pathogenic *Leptospira* DNA in 16S rDNA real-time qPCR and resulted positive, with the filter containing 100 times more leptospires than the filtered water. Therefore, this method of pretreatment was applied to the water samples collected.

DNA was extracted from 100 µL of pretreated urine samples and from 100 µL of EDTA-treated blood samples using the ID Gene^®^ Mag Universal Extraction Kit (IDvet, Grabels, France) on the KingFisher™ Flex Purification System (Life Technologies, Carlsbad, CA, USA) platform. After a pretreatment with 2.5 µL of lysozyme (Sigma-Aldrich, St. Louis, MO, USA) at 37 °C for 15 min, the extraction was performed in accordance with the manufacturer’s instructions for the extraction of bacteria. Given the poor cellular matrices, 20 µg of a poly-A carrier (Sigma-Aldrich, St. Louis, MO, USA) was added to each urine sample to increase the recovery efficiency of nucleic acids.

Two hundred and fifty milliliters of refrigerated water from each site identified as a possible source of contamination was pretreated as mentioned above. Half the filter was cut into small pieces with sterile scissors and transferred to a 2 mL collection tube containing 400 µL of sterile PBS. Both 400 µL of filtered water and the cut filter were subjected to extraction with the QIAamp DNA Mini kit (Qiagen, Hilden, Germany), following the manufacturer’s instructions for swabs.

All DNA extractions included sterile PBS, as a negative process control. Extracted DNA was stored at −20 °C, until the amplification step.

### 2.4. Real-Time qPCR Detection

DNA extracts from biological and water samples were subjected to a TaqMan-based real-time qPCR assay detecting only pathogenic *Leptospira* strains and targeting an 87 bp fragment, corresponding to a portion of the gene encoding the 16S rDNA, using primers reported in Supplementary Table S1 [16]. The amplification was performed in a 25 µL final volume, containing 3 µL of extracted DNA, 12.5 µL of 2X Path-ID™ qPCR Master Mix (Thermo Fisher Scientific, Waltham, MA, USA), 300 nM of each primer, and 100 nM of a 5′ FAM probe. All the amplification assays included a negative control (nuclease-free water), a negative bacterial genomic control (DNA of *L. biflexa* serovar *Patoc*), and a positive control (DNA of *L. interrogans* serovar Icterohaemorrhagiae). The assay was performed on Applied Biosystems real-time PCR instruments (7900HT Fast Real-time and QuantStudio 5, Thermo Fisher Scientific, Waltham, MA, USA) with the following thermal conditions: a hot-start step of 95 °C for 10 min, and 40 cycles of 95 °C for 15 s and 60 °C for 60 s. Samples with cycle threshold (Ct) < 38 were considered positive. Samples with Ct values ranging between 38 and 40 were considered doubtful, whereas samples having no FAM fluorescence signal or with Ct > 40 were considered negative.

### 2.5. Genotyping by Multi-Locus Sequence Typing (MLST) Analysis

Multi-locus sequence typing (MLST) analysis was applied to each DNA sample that tested positive for leptospiral DNA in the real-time qPCR. If more than one biological matrix was available for the same dog, the analysis was attempted on all the samples to maximize the probability of success. To genotype leptospires, the scheme proposed by Boonsilp in 2013 [17] and the protocol adopted by Weiss and colleagues [18] were used, as previously described [19,20], using primers reported in Supplementary Table S1. The scheme was based on the seven housekeeping genes: UDP-N-acetylglucosamine pyrophosphorylase (*glmU*), NAD(P)(+) transhydrogenase alpha subunit (*pntA*), 2-oxoglutarate dehydrogenase E1 component (*sucA*), triosephosphate isomerase (*tpiA*), 1-phosphofructokinase (*pfkB*), rod shape-determining protein rodA (*mreA*), and acyl-CoA transferase/carnitine dehydratase (*caiB*).

Nucleotide sequences for each of the seven genes were concatenated (final sequence of 3111 nucleotides) and aligned with reference strains from PubMLST [21] and the Italian Reference Centre for Animal Leptospirosis (IZSLER, Brescia, Italy) databases using BioEdit software. A phylogenetic analysis was conducted in MEGA 11, version 10.1.7, using the Neighbor-Joining method and the Maximum Composite Likelihood model, with a bootstrap analysis based on 1000 replicates [22,23,24,25].

## 3. Results

### 3.1. Data on Animals Sampled and Enrolled in the Study

Among the study population, 40/59 (67.8%) dogs were males and 19/59 (32.2%) were females. The median age of all dogs was four years (range <1–15 years). Of the 59 dogs, 11 (18.6%) were purebred and 48 (81.4%) were mixed breed. Thirty (50.9%) dogs were regularly vaccinated against leptospirosis: twenty-four with tetravalent vaccine (Nobivac L4, MSD Animal Health, Madison, NJ, USA) containing *Leptospira interrogans* serogroup Canicola serovar Portland-vere, serogroup Icterohaemorrhagiae serovar Copenhageni, serogroup Australis serovar Bratislava, and *L. kirschneri* serogroup Grippotyphosa serovar Dadas, and six with bivalent vaccine (Canigen L, Virbac, Carros, France) containing *Leptospira interrogans* serogroup Canicola serovar Canicola and serogroup Icterohaemorrhagiae serovar Icterohaemorrhagiae. In 9/30 regularly vaccinated dogs, the vaccine was administered less than 15 weeks before the sampling. In the remaining 29/59 (49.1%) dogs, the bivalent *Leptospira* vaccine was administered more than a year before the testing. The 3 ill dogs were mixed-breed males, aged between 4 and 15 years, of which 2 were regularly vaccinated (with bivalent vaccine) and 1 was vaccinated more than a year ago.

Soon after the identification of the first cases of canine leptospirosis, the territorial veterinary health authority implemented the following prescriptions: (i) separation between infected and healthy dogs, (ii) antimicrobial treatment of infected and potentially infected dogs (direct contacts and sharing environment), (iii) daily disinfection of the boxes with 10% Amukine^®^ (sodium hypochlorite) and floor drying in the absence of dogs, (iv) rodent control, and (v) feed storage in closed containers. The three infected dogs showing clinical signs of leptospirosis were hospitalized for medical treatment consisting of administration of fluid therapy under continuous intravenous infusion and amoxicillin and clavulanic acid (20 mg/kg IV q8h) for 14 days. All dogs recovered from the disease. The dogs not showing clinical manifestations, but which tested positive for *Leptospira* spp. infection by serologic and molecular assays, and the subjects that came in close contact with positive dogs, were treated with doxycycline (10 mg/kg PO q24h) for 14 days.

### 3.2. Detection of Leptospiral Infection Using MAT and qPCR Assays

The sera of all 59 dogs included in the study were tested by the MAT assay and 32/59 (54.2%) dogs tested positive to antibodies against at least one of the pathogenic *Leptospira* serovars included in the antigen panel of the MAT assay, with a cut-off ≥ 1:100 (Table 2). Twenty-four of the positive dogs (24/32) had MAT titers ≥ 1:100 against non-vaccine serogroups or ≥1:800 against vaccine serogroups and were recognized as of potential infectious origin. Thirteen of these were regularly vaccinated, four received the vaccine less than fifteen weeks before the sampling, and twenty-two (37.3% of the dogs included in the study) exhibited seropositivity against at least one of the three serovars belonging to the Sejroe serogroup included in the MAT antigen panel (Hardjo, Saxkoebing, and Sejroe). Eight of the positive dogs had MAT titers < 1:800 against vaccine serogroups only and were recognized as of potential vaccine origin. Seven of these were regularly vaccinated and four received the vaccine less than fifteen weeks before the sampling. In most of the seropositive dogs, multiple titers against different serovars and serogroups were detected.

EDTA-treated blood and/or urine samples of all the 59 dogs included in the study were tested by the qPCR assay, and 10/59 (17%) dogs were positive to leptospiral DNA (Table 2): 2 dogs tested positive in the blood sample only, 6 dogs in the urine sample only, and 2 dogs in both blood and urine samples. Two dogs (ID: 57878 and 58142) that tested positive for leptospiral DNA showed MAT antibody titers presumably recognized as of vaccine origin, four dogs (ID: 58152, 58161, 58163, and 58166) showed MAT antibody titers presumably recognized as of infectious origin, and four dogs (ID: 57880, 58043, 58121, and 58156) were negative in the MAT assay.

In total, leptospiral infection was suspected in 30/59 (50.9%) dogs, including the 3 dogs showing clinical manifestations referable to leptospirosis: 20 had MAT titers presumably recognized as of infectious origin only, 4 had leptospiral DNA in blood and/or urine samples only, 4 had both MAT titers presumably recognized as of infectious origin and leptospiral DNA in samples tested, and 2 had MAT titers presumably recognized as of vaccine origin but were positive in the qPCR (Table 2). The dogs that tested positive were distributed throughout the kennel facility and included subjects who never came into contact with each other. Twenty-one of the thirty (70%) infection-suspected dogs were males and nine (30%) were females. The median age of the 30 infection-suspected dogs was 4 years (range <1–15 years). Three of the thirty (10%) dogs were purebred and twenty-seven (90%) were mixed breed. Seventeen of the thirty (56.7%) dogs were regularly vaccinated against leptospirosis: fourteen with tetravalent vaccine and three with bivalent vaccine.

### 3.3. Molecular Detection of Leptospira *spp.* DNA in Well Water

No DNA belonging to pathogenic *Leptospira* spp. was detected in the water of the well that served the kennel.

### 3.4. Genotyping by MLST Analysis

Samples of the ten dogs that tested positive to leptospiral DNA were submitted for MLST analysis. A complete MLST profile was obtained from one dog (ID: 58152), while for three dogs a partial profile was defined (ID: 57878, 57880, and 58043) (Table 3). Nucleotide sequences were submitted to the GenBank database under the following accession numbers: OM287411–OM287417 and OM302511–OM302527. All four infecting *Leptospira* belonged to ST155. We were unable to achieve a successful PCR amplification in MLST loci in the samples from the remaining six dogs, probably due to the low amount of leptospiral DNA present.

**Figure 1:**
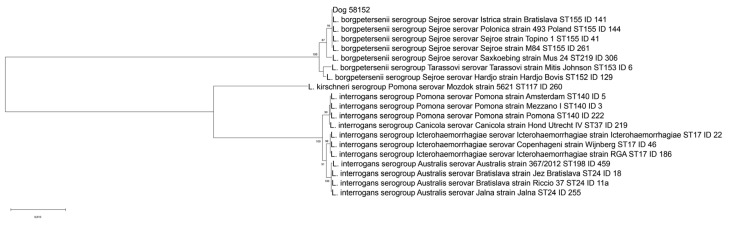
Phylogenetic tree built on concatenated sequences of the seven multi-locus sequence typing (MLST) loci (3111 bp) of the scheme proposed by Boonsilp and colleagues [17]. Phylogeny was conducted in MEGA 11 using the Neighbor-Joining method and bootstrap values are indicated on the respective branches. The samples are indicated with their ID, which represents a unique identification number of the relative reference strain present in the collection of the Italian Reference Centre for Animal Leptospirosis (IZSLER, Brescia, Italy).

*Leptospira* ST155 clustered with strains characterized as *L. borgpetersenii* serogroup Sejroe (Figure 1), while the serovar status was not deducible with certainty from the MLST profile. Nevertheless, *Leptospira* ST155 could be associated with reference strains characterized as serovars Sejroe, Polonica, and Istrica from PubMLST and the Italian Reference Centre for Animal Leptospirosis (IZSLER, Brescia, Italy) databases, but not to serovars Hardjo and Saxkoebing, which were characterized as ST152 and ST219, respectively.

## 4. Discussion

In the investigated kennel, 3 dogs showed clinical manifestations referable to leptospirosis and 30/59 (50.8%) dogs (the 3 ill dogs and 27 apparently healthy dogs) had MAT titer and/or molecular positivity indicative of *Leptospira* infection. Among them, 22 (37.3% of the dogs included in the study) exhibited seropositivity against at least 1 of the 3 serovars belonging to the Sejroe serogroup included in the MAT antigen panel (Hardjo, Saxkoebing, and Sejroe). Previous studies conducted in Italy in kennels investigated the diffusion of leptospiral infection in clinically healthy dogs only and reported variable seroprevalence values ranging from 12.7% to 49.2% [7,11]. In particular, Scanziani and colleagues reported high seroprevalence of leptospiral infection in kennel dogs regardless of vaccination status and suggested that low levels of hygiene were associated to a higher spread of infection [7].

The high number of infected dogs, some of which showed clinical signs referable to leptospirosis, is attributable to a recent outbreak in dogs with no history of previous cases of leptospirosis. The high MAT titers against a single *Leptospira* serogroup and the detection of *Leptospira* DNA both in seronegative dogs and in dogs showing low MAT titers are indicative of acute infections due to the same *Leptospira* variant. Unfortunately, it was not possible to perform a second MAT test on the positive dogs to evaluate seroconversion and to verify the acute nature of the infection [3]. Nevertheless, the usefulness of the molecular analysis, both on blood and urine samples, to identify infected subjects not detected by the MAT test is evident and confirmed [3,26].

The majority of the dogs (22/24) showing MAT titers presumably recognized as of infectious origin exhibited seropositivity against at least one of the three serovars belonging to the Sejroe serogroup included in the MAT antigen panel adopted in this study (Hardjo, Saxkoebing, and Sejroe). To date, Sejroe serogroup infection in dogs was sporadically reported in dogs and it has rarely been associated with clinical manifestations [4,7,12,19,27,28].

MLST analysis confirmed that the infection was sustained by *L. borgpetersenii* serogroup Sejroe (*Leptospira* ST155). From the comparison with reference strains, it is possible to assume that the identified *Leptospira* belongs to one of the Sejroe, Polonica, or Istrica serovars. The detection of *Leptospira* ST155 in regularly vaccinated dogs showing clinical signs referable to leptospirosis was already reported by Bertasio and colleagues in 2020 [19]. The same *Leptospira* ST has previously also been identified in mice [19] and hedgehogs (Italian Reference Centre for Animal Leptospirosis, Brescia, Italy, database) in the Veneto region, Italy, which may represent its maintenance hosts. Further studies are needed to confirm this hypothesis, particularly on the role of mice that appear to be involved in maintaining serogroup Sejroe in the environment [29,30].

From the available data, it is not possible to assess whether *Leptospira* was introduced into the kennel through external maintenance hosts, such as rodents, or through the entry of an infected dog. However, since the dogs that tested positive were housed throughout the kennel facility and included subjects who never came into contact with each other, the circulation of infected rodents appears more realistic, rather than the entry of an infected dog that would have presumably generated an outbreak localized in an area of the kennel, infecting only the subjects in contact. The hypothesis that dogs were infected through contaminated well water was evaluated, investigating the presence of leptospiral DNA in several water samples, but they tested negative. This finding would lead to excluding any contamination of the water by reservoir hosts, but several studies have reported increased human exposure days after heavy rain events, putting leptospires kept viable in soils in suspension [31]. Therefore, the possibility that *Leptospira* ST155 infected dogs after heavy rains through contaminated water a few weeks before the outbreak cannot be ruled out with absolute certainty.

The majority (56.7%) of dogs that tested positive in this study were regularly vaccinated against leptospirosis with tetravalent or bivalent vaccines, including two dogs showing clinical manifestations referable to leptospirosis. Since both vaccines administered do not include antigens from serovars belonging to the Sejroe serogroup, the infections caused by this serogroup may have eluded the immune response evoked by vaccination. As previously reported for the widespread use of the bivalent vaccine against Canicola and Icterohaemorrhagiae serovars, which led to the increased reporting of other serovars belonging to the Grippotyphosa and Australis serogroups in dogs [4,5], it is possible to expect future evolutions in the epidemiology of *Leptospira* infection, due to the adoption of the tetravalent vaccines, making the circulation of some other serovars in dogs more evident.

Although MAT is a serogroup rather than a serovar-specific test [2], the panel of serovars tested should ideally be defined based on serological prevalence data in the geographic location investigated [3], because different responses are detectable between serovars belonging to the same serogroup. Particularly, the MAT antigen panel commonly adopted in our geographical area included only the serovar Hardjo of the serogroup Sejroe, potentially resulting in an underestimation of the circulation of other serovars belonging to this serogroup in the dog population [7,10,12]. Furthermore, a recent study conducted in dogs in north-eastern Italy evidenced the circulation of different serovars belonging to the Sejroe serogroup [19], and the MAT antigen panel has been expanded accordingly by adding the Sejroe and Saxkoebing serovars to the pre-existing Hardjo. The expanded MAT antigen panel used in this study allowed us to identify a higher number of seropositive dogs (two dogs showed antibodies against Sejroe or Saxkoebing serovars but were negative to Hardjo), proving its usefulness in diagnosing canine leptospirosis in our area.

Dogs play an important role in the epidemiology of *Leptospira* infection as they can act as both incidental and maintenance hosts with or without clinical symptoms, shedding leptospires in their urine [32]. With the exception of the *Leptospira interrogans* serovar Canicola, for which the dog represents the main maintenance host, and serovars belonging to the Australis serogroup which could also be maintained in these animals, dogs are assumed to be incidental hosts for the infecting serovar and, consequently, shedding is likely to be brief when compared to that of reservoir hosts such as rodents [3,4]. Therefore, the zoonotic role of infected dogs is minor compared to that played by rodents. In this regard, no or uncommon serological reactivity against *Leptospira* serovars was reported among veterinary staff using adequate personal protections or pet owners exposed to dogs with acute leptospirosis [33,34], and Vitale and colleagues recently reported two cases of human leptospiral infection in Sicily (Italy) related to contact with water or greens cultivated along a riverbank and not to infected dogs [35]. Furthermore, in Italy between 1994 and 1996, the majority of human leptospirosis cases originated from contact with contaminated water rather than by direct contact with animals, and rodents were implicated in 50% of cases involving direct contact with animals [36]. However, dogs represent a significant connection between wildlife and humans and can serve as indicators of the presence of leptospires in specific environments [3]. The present study corroborates the role played by the dog as an environmental sentinel species. In the investigated kennel, the early identification of the infected dogs in a specific high-risk environment for the transmission of leptospirosis both to animals and humans allowed to promptly control the outbreak, to act on the presence of any maintenance hosts, and to prevent human disease.

There are some limitations in this study. Some intrinsic limitations of the MAT assay can lead to misinterpretations of the obtained results, because this test cannot definitely discriminate between vaccinated and infected dogs. Indeed, cross-reaction between different serovars and paradoxical reaction are common events [1,3], infected dogs can be serological-negative during the acute phase of the infection [1,3], antibodies against leptospiral serogroups not included in the MAT panel are not detected, and high post-vaccine/post-infection titers (≥1:800) can persist for long periods (15 weeks or up to more than a year) after vaccinations with concomitant cross-reactivity to non-vaccine serogroups [37,38]. In this study, three dogs (ID: 58037, 58124, and 58168) with MAT titers attributed to natural infectious origin according to the criteria adopted in this study may not have an active infection because they reported higher titers against vaccine serogroups [39]. Moreover, these dogs included the two subjects (ID: 58037 and 58168) which did not show seropositivity against the Sejroe serogroup. These two dogs also had MAT titers against serogroups not included in the vaccines and may have been naturally infected with leptospires other than ST155 within or before entering the kennel. Finally, it was not possible to collect and test mice and rats circulating in the kennel to evaluate their role as maintenance hosts for the identified *Leptospira* variant. However, the absence of new clinical cases reported after rodent control supports the hypothesis that these animals acted as the source of infection for dogs.

## 5. Conclusions

Dogs are susceptible to *Leptospira* infections and can develop severe and potentially fatal clinical manifestations. However, humans contract leptospirosis mainly from the same environmental sources of infection to which dogs are exposed (e.g., water, soil, rodents), rather than from direct contact with the dog or fomites contaminated by its urine. For this reason, the dog can represent an important sentinel species as well as a potential reservoir host. The role as a sentinel species is crucial, especially in high-risk environments such as kennels, where contact between humans, dogs, rodents, and synanthropic or wild species is frequent. Therefore, the surveillance of *Leptospira* infection in kennels is strongly recommended, even when the correct vaccine prophylaxis is administered, because the vaccines currently available are not able to protect from all the serogroups.

## Figures and Tables

**Table 1:** Panel of eleven *Leptospira* spp. used as live antigens for the MAT assay.

Species	Serogroup	Serovar	Strain
*L. interrogans*	Canicola	Canicola	Alarik n.2
*L. interrogans*	Icterohaemorrhagiae	Copenhageni	Wijnberg n.1
*L. interrogans*	Icterohaemorrhagiae	Icterohaemorrhagiae	Bianchi
*L. interrogans*	Australis	Bratislava	Riccio 2 n.47
*L. kirschneri*	Grippotyphosa	Grippotyphosa	Moskva V n.54
*L. interrogans*	Pomona	Pomona	Pomona n.222
*L. borgpetersenii*	Tarassovi	Tarassovi	Mitis-Johnson n.6
*L. borgpetersenii*	Ballum	Ballum	Mus 127 n.217
*L. interrogans*	Sejroe	Hardjo	Hadjoprajitno n.224
*L. borgpetersenii*	Sejroe	Sejroe	M84
*L. borgpetersenii*	Sejroe	Saxkoebing	Mus24

**Table 2:** Dogs that tested positive by MAT and/or qPCR assays.

Dogs	Vaccination ^1^	Clinical Manifestations	Ca-Ca	Ic-Co	Ic-Ic	Au-Br	Gr-Gr	Po-Po	Ta-Ta	Ba-Ba	Se-Ha	Se-Se	Se-Sa	Type ^2^	qPCR ^3^
57878	R-L4	yes	0	100	100	0	0	0	0	0	0	0	0	vaccine	BU
58038	R-L4	no	0	200	200	0	0	0	0	0	0	0	0	vaccine	
58040	N-L2	no	200	400	200	0	0	0	0	0	0	0	0	vaccine	
58129	R-L4*	no	0	200	0	100	400	0	0	0	0	0	0	vaccine	
58135	R-L4*	no	200	200	0	100	400	0	0	0	0	0	0	vaccine	
58142	R-L4*	no	100	100	0	0	0	0	0	0	0	0	0	vaccine	B
58148	R-L4	no	100	0	0	0	0	0	0	0	0	0	0	vaccine	
58171	R-L4*	no	100	200	200	0	400	0	0	0	0	0	0	vaccine	
57883	N-L2	no	0	0	0	0	0	0	0	0	200	200	200	infection	
58037	R-L4*	no	0	0	0	100	400	100	0	0	0	0	0	infection	
58039	R-L4	no	0	0	0	0	0	0	0	0	0	400	400	infection	
58041	R-L4	no	200	400	800	0	200	100	0	100	400	800	800	infection	
58042	N-L2	no	200	200	0	200	400	100	0	0	800	400	400	infection	
58044	R-L4*	no	100	0	0	400	200	0	0	0	400	800	400	infection	
58124	R-L4	no	200	400	400	0	0	0	0	0	0	200	0	infection	
58127	N-L2	no	0	0	0	0	0	0	0	0	400	400	400	infection	
58128	R-L4	no	0	0	0	0	0	0	0	0	200	200	100	infection	
58131	N-L2	no	0	0	0	0	0	0	0	0	1600	1600	800	infection	
58132	N-L2	no	0	0	0	0	0	0	0	0	3200	800	800	infection	
58133	R-L4*	no	400	0	0	0	100	0	0	0	1600	1600	1600	infection	
58139	N-L2	no	0	0	0	0	0	0	0	0	100	0	0	infection	
58141	N-L2	no	0	0	0	0	0	0	0	0	100	800	400	infection	
58144	N-L2	no	0	0	0	0	0	0	0	100	200	200	400	infection	
58146	N-L2	no	0	0	0	0	0	0	0	0	200	800	400	infection	
58152	R-L2	no	0	0	0	100	0	0	0	0	800	800	400	infection	U
58155	R-L2	no	0	0	0	0	0	0	0	0	400	0	0	infection	
58161	R-L2	no	0	0	0	0	0	0	0	0	200	200	200	infection	U
58163	R-L4	no	0	0	0	0	0	0	0	0	800	800	400	infection	U
58166	R-L4	no	0	0	0	0	0	100	0	0	800	800	1600	infection	U
58168	R-L4*	no	400	0	0	200	800	400	0	0	0	0	0	infection	
58173	N-L2	no	0	100	0	0	0	0	0	0	200	400	200	infection	
60311	N-L2	yes	0	0	0	0	0	0	0	0	400	3200	1600	infection	
57880	R-L4	no	0	0	0	0	0	0	0	0	0	0	0	negative	B
58043	R-L4	yes	0	0	0	0	0	0	0	0	0	0	0	negative	BU
58121	N-L2	no	0	0	0	0	0	0	0	0	0	0	0	negative	U
58156	N-L2	no	0	0	0	0	0	0	0	0	0	0	0	negative	U

Ca-Ca: Canicola–Canicola, Ic-Co: Icterohaemorrhagiae–Copenhageni, Ic-Ic: Icterohaemorrhagiae–Icterohaemorrhagiae, Au-Br: Australis–Bratislava, Gr-Gr: Grippotyphosa–Grippotyphosa, Po-Po: Pomona–Pomona, Ta-Ta: Tarassovi–Tarassovi, Ba-Ba: Ballum–Ballum, Se-Ha: Sejroe–Hardjo, Se-Se: Sejroe–Sejroe, Se-Sa: Sejroe–Saxkoebing. ^1^ R-L4: Regularly vaccinated with tetravalent vaccine. R-L4*: Regularly vaccinated with tetravalent vaccine less than 15 weeks before the sampling. R-L2: Regularly vaccinated with bivalent vaccine. N-L2: Not regularly vaccinated with bivalent vaccine. ^2^ Positive titers ≥ 1:100 against non-vaccine serogroups or ≥ 1:800 against vaccine serogroups (Canicola and Icterohaemorrhagiae for bivalent vaccine, and Canicola, Icterohaemorrhagiae, Australis, and Grippotyphosa for tetravalent vaccine) were recognized as of potential infectious origin (infection). Positive titers < 1:800 against vaccine serogroups only were recognized as of potential vaccine origin (vaccine). ^3^ B: Dogs tested positive to *Leptospira* DNA in EDTA-treated blood sample. U: Dogs tested positive to *Leptospira* DNA in urine sample.

**Table 3:** Results of multi-locus sequence typing (MLST) analysis.

ID	ST	glmU	pntA	sucA	tpiA	pfkB	mreA	caiB
57878	155 (partial)	24	n.d.	36	34	n.d.	27	n.d.
58152	155	24	28	36	34	37	27	28
57880	155 (partial)	24	n.d.	36	34	n.d.	27	28
58043	155 (partial)	24	28	36	34	37	n.d.	28

ST: sequence type; n.d.: not defined.

## Data Availability

The datasets supporting the conclusions of this article are included within the article. The bacterial nucleotide sequences obtained in this study are openly available in the GenBank database (https://www.ncbi.nlm.nih.gov/genbank/, accessed on 1 February 2022; ID: OM287411-OM287417 and OM302511-OM302527).

## Supplementary Materials

The following supporting information can be downloaded at: https://www.mdpi.com/article/10.3390/ijerph19073906/s1, Table S1: primers and probes used in the real-time qPCR and end-point PCR reactions.

## References

[1] Sykes J.E., Hartmann K., Lunn K.F., Moore G.E., Stoddard R.A., Goldstein R.E. (2011). 2010 ACVIM small animal consensus statement on leptospirosis: Diagnosis, epidemiology, treatment, and prevention. J. Vet. Intern. Med..

[2] Levett P.N. (2001). Leptospirosis. Clin. Microbiol. Rev..

[3] Schuller S., Francey T., Hartmann K., Hugonnard M., Kohn B., Nally J.E., Sykes J. (2015). European consensus statement on leptospirosis in dogs and cats. J. Small Anim. Pract..

[4] Ellis W.A. (2010). Control of canine leptospirosis in Europe: Time for a change?. Vet. Rec..

[5] André-Fontaine G. (2006). Canine leptospirosis—Do we have a problem?. Vet. Microbiol..

[6] Jull D.J., Heath K.R. (1960). The evaluation of a combined *L. canicola* and *L. icterohaemorrhagiae* vaccine on hamsters and dogs. J. Small Anim. Pract..

[7] Scanziani E., Origgi F., Giusti A.M., Iacchia G., Vasino A., Pirovano G., Scarpa P., Tagliabue S. (2002). Serological survey of leptospiral infection in kennelled dogs in Italy. J. Small Anim. Pract..

[8] Klaasen H.L., van der Veen M., Molkenboer M.J., Sutton D. (2013). A novel tetravalent *Leptospira* bacterin protects against infection and shedding following challenge in dogs. Vet. Rec..

[9] Klaasen H.L., van der Veen M., Sutton D., Molkenboer M.J. (2014). A new tetravalent canine leptospirosis vaccine provides at least 12 months immunity against infection. Vet. Immunol. Immunopathol..

[10] Bertelloni F., Cilia G., Turchi B., Pinzauti P., Cerri D., Fratini F. (2019). Epidemiology of leptospirosis in North-Central Italy: Fifteen years of serological data (2002–2016). Comp. Immunol. Microbiol. Infect. Dis..

[11] Piredda I., Ponti M.N., Piras A., Palmas B., Pintore P., Pedditzi A., Chisu V. (2021). New insights on *Leptospira* infections in a canine population from North Sardinia, Italy: A sero-epidemiological study. Biology.

[12] Tagliabue S., Figarolli B.M., D’Incau M., Foschi G., Gennero M.S., Giordani R., Natale A., Papa P., Ponti N., Scaltrito D. (2016). Serological surveillance of Leptospirosis in Italy: Two-year national data (2010–2011). Vet. Ital..

[13] Day M.J., Horzinek M.C., Schultz R.D., Squires R.A., Vaccination Guidelines Group (VGG) of the World Small Animal Veterinary Association (WSAVA) (2016). WSAVA Guidelines for the vaccination of dogs and cats. J. Small. Anim. Pract..

[14] Leptospirosis. OIE Manual of Diagnostic Tests and Vaccines for Terrestrial Animals. World Organisation for Animal Health..

[15] Urban L., Holzer A., Baronas J.J., Hall M.B., Braeuninger-Weimer P., Scherm M.J., Kunz D.J., Perera S.N., Martin-Herranz D.E., Tipper E.T. (2021). Freshwater monitoring by nanopore sequencing. eLife.

[16] Smythe L.D., Smith I.L., Smith G.A., Dohnt M.F., Symonds M.L., Barnett L.J., McKay D.B. (2002). A quantitative PCR (TaqMan) assay for pathogenic *Leptospira* spp.. BMC Infect. Dis..

[17] Boonsilp S., Thaipadungpanit J., Amornchai P., Wuthiekanun V., Bailey M.S., Holden M.T., Zhang C., Jiang X., Koizumi N., Taylor K. (2013). A single multilocus sequence typing (MLST) scheme for seven pathogenic *Leptospira* species. PLoS Negl. Trop. Dis..

[18] Weiss S., Menezes A., Woods K., Chanthongthip A., Dittrich S., Opoku-Boateng A., Kimuli M., Chalker V. (2016). An extended multilocus sequence typing (MLST) scheme for rapid direct typing of *Leptospira* from clinical samples. PLoS Negl. Trop. Dis..

[19] Bertasio C., Boniotti M.B., Lucchese L., Ceglie L., Bellinati L., Mazzucato M., Furlanello T., D’Incau M., Natale A. (2020). Detection of new *Leptospira* genotypes infecting symptomatic dogs: Is a new vaccine formulation needed?. Pathogens.

[20] Bertasio C., Papetti A., Scaltriti E., Tagliabue S., D’Incau M., Boniotti M.B. (2020). Serological survey and molecular typing reveal new *Leptospira* serogroup Pomona strains among pigs of Northern Italy. Pathogens.

[21] Leptospira spp. MLST Database. https://pubmlst.org/leptospira/.

[22] Saitou N., Nei M. (1987). The neighbor-joining method: A new method for reconstructing phylogenetic trees. Mol. Biol. Evol..

[23] Stecher G., Tamura K., Kumar S. (2020). Molecular evolutionary genetics analysis (MEGA) for macOS. Mol. Biol. Evol..

[24] Tamura K., Nei M., Kumar S. (2004). Prospects for inferring very large phylogenies by using the neighbor-joining method. Proc. Natl. Acad. Sci. USA.

[25] Tamura K., Stecher G., Kumar S. (2021). MEGA11: Molecular evolutionary genetics analysis version 11. Mol. Biol. Evol..

[26] Troìa R., Balboni A., Zamagni S., Frigo S., Magna L., Perissinotto L., Battilani M., Dondi F. (2018). Prospective evaluation of rapid point-of-care tests for the diagnosis of acute leptospirosis in dogs. Vet. J..

[27] Rühl-Fehlert C.I., Brem S., Feller W., Kopp H., Meyer P., Rinke M. (2000). Clinical, microbiological and pathological observations in laboratory beagle dogs infected with leptospires of the serogroup Sejroe. Exp. Toxicol. Pathol..

[28] Scanziani E., Crippa L., Giusti A.M., Luini M., Pacciarini M.L., Tagliabue S., Cavalletti E. (1995). *Leptospira interrogans* serovar sejroe infection in a group of laboratory dogs. Lab. Anim..

[29] Alston J.M., Broom J.C. (1958). Leptospirosis in Man and Animals.

[30] Little T.W.A., Ellis W.A., Little T.W.A. (1986). Changes in our understanding of the epidemiology of leptospirosis. The Present State of the Art of Letpospirosis Diagnosis and Control.

[31] Bierque E., Thibeaux R., Girault D., Soupé-Gilbert M.E., Goarant C. (2020). A systematic review of *Leptospira* in water and soil environments. PLoS ONE.

[32] Rojas P., Monahan A.M., Schuller S., Miller I.S., Markey B.K., Nally J.E. (2010). Detection and quantification of leptospires in urine of dogs: A maintenance host for the zoonotic disease leptospirosis. Eur. J. Clin. Microbiol. Infect. Dis..

[33] Barmettler R., Schweighauser A., Bigler S., Grooters A.M., Francey T. (2011). Assessment of exposure to *Leptospira* serovars in veterinary staff and dog owners in contact with infected dogs. J. Am. Vet. Med. Assoc..

[34] Mazzotta E., Lucchese L., Salata C., Furlanello T., Baroni E., Zotti A., Venturi G., Fincato A., Marchione S., Capello K. (2022). Are small animal practitioners occupationally exposed to leptospirosis? Results of a serological survey. Int. J. Environ. Res. Public. Health..

[35] Vitale M., Agnello S., Chetta M., Amato B., Vitale G., Bella C.D., Vicari D., Presti V.D.M.L. (2018). Human leptospirosis cases in Palermo Italy. The role of rodents and climate. J. Infect. Public. Health..

[36] Ciceroni L., Stepan E., Pinto A., Pizzocaro P., Dettori G., Franzin L., Lupidi R., Mansueto S., Manera A., Ioli A. (2000). Epidemiological trend of human leptospirosis in Italy between 1994 and 1996. Eur. J. Epidemiol..

[37] Barr S.C., McDonough P.L., Scipioni-Ball R.L., Starr J.K. (2005). Serologic responses of dogs given a commercial vaccine against *Leptospira interrogans* serovar pomona and *Leptospira kirschneri* serovar grippotyphosa. Am. J. Vet. Res..

[38] Martin L.E., Wiggans K.T., Wennogle S.A., Curtis K., Chandrashekar R., Lappin M.R. (2014). Vaccine-associated *Leptospira* antibodies in client-owned dogs. J. Vet. Intern. Med..

[39] Kohn B., Steinicke K., Arndt G., Gruber A.D., Guerra B., Jansen A., Kaser-Hotz B., Klopfleisch R., Lotz F., Luge E. (2010). Pulmonary abnormalities in dogs with leptospirosis. J. Vet. Intern. Med..

