# Supplementary material for: Outbreak of Leptospira borgpetersenii Serogroup Sejroe Infection in Kennel: The Role of Dogs as Sentinel in Specific Environments
Source: Int J Environ Res Public Health. 2022 Mar 25;19(7):3906. doi: 10.3390/ijerph19073906 (PMC8997430; doi:10.3390/ijerph19073906)
Supplement: Supplementary file 1 [file ijerph-19-03906-s001.zip › ijerph-1633085-supplementary.pdf]

Table S1. Primers and probes used in the real-time qPCR and end-point PCR reactions.

Assay	Gene	Primers and Probes	Sequence	Amplicon	Reference
qPCR	*rrs* (16S)	Lepto F	5'-CCCGCGTCCGATTAG-3'	87 bp	16
		Lepto R	5'-TCCATTGTGGCCGRACAC-3'		
		probe	(FAM)5'-CTCACCAAGGCGACGATCGGTAGC-3'(TAMRA)		
nestedPCR	*glmU*	glmU-FM	AGGATAAGGTCGCTGTGGTA	650 bp	17
		glmU-RM	AGTTTTTTTCCGGAGTTTCT		
		1-glmU-2F_M13	TGTAAAACGACGGCCAGT CGYATGAAAACGGATCAG	598 bp	18
		1-glmU-2R_M13	CAGGAAACAGCTATGACC GGAAGRTARTATTCDCCCTG		
nestedPCR	*pntA*	pntA-FM	TAGGAAARATGAAACCRGGAAC	621 bp	17
		pntA-RM	AAGAAGCAAGATCCACAAYTAC		
		2-pntA-2F_M13	TGTAAAACGACGGCCAGT ATTTATYTVGGRATGTTYCA	607 bp	18
		2-pntA-2R_M13	CAGGAAACAGCTATGACC GATTTCATRTTATCYACRAT		
nestedPCR	*sucA*	sucA-FM	TCATTCCACTTYTAGATACGAT	640 bp	17
		sucA-RM	TCTTTTTTGAATTTTTGACG		
		3-sucA-2F_M13	TGTAAAACGACGGCCAGT GCSGGTRATCATCWBATGG	552 bp	18
		3-sucA-2R_M13	CAGGAAACAGCTATGACC GRAAWCCYTTYGCAAGATC		
nestedPCR	*tpiA*	tpiA-FM	TTGCAGGAAACTGGAAAATGAAT	639 bp	17
		tpiA-RM	GTTTTACRGAACCHCCGTAGAGAAT		
		4-tpiA-2F_M13	TGTAAAACGACGGCCAGT ATTTCYTTACGAATRAAAGARTG	555 bp	18
		4-tpiA-2R_M13	CAGGAAACAGCTATGACC CMCATTCGATYMRAGAAAA		
nestedPCR	*pfkB*	pfkB-FM	CGGAGAGTTTTATAARAAGGACAT	588 bp	17
		pfkB-RM	AGAACACCCGCCGCAAAACAAT		
		5-pfkB-2F_M13	TGTAAAACGACGGCCAGT GTYGTATCGATCGSYTTC	540 bp	18
		5-pfkB-2R_M13	CAGGAAACAGCTATGACC YYCCSGAAGAYAASGGWCAT		
nestedPCR	*mreA*	mreA-FM	GGCTCGCTCTYGACGGAAA	719 bp	17
		mreA-RM	TCCRTAACTCATAAAMGACAAAGG		
		6-mreA-2F_M13	TGTAAAACGACGGCCAGT CRRGAAGYRGTGGATCAGG	568 bp	18
		6-mreA-2R_M13	CAGGAAACAGCTATGACC CKATCCTTACTYTCRTARCT		
nestedPCR	*caiB*	caiB-F	CAACTTGCGGAYATAGGAGGAG	650 bp	17
		caiB-R	ATTATGTTCCCCGTGAYTCG		
		7-caiB-2F_M13	TGTAAAACGACGGCCAGT CTTKCTTCRATYTTGGCG	589 bp	18
		7-caiB-2R_M13	CAGGAAACAGCTATGACC AMCGATATGTWAYMGGRGTT		
